# Temperature effect on a weighted vortex spin-torque nano-oscillator for neuromorphic computing

**DOI:** 10.1038/s41598-024-60929-3

**Published:** 2024-05-02

**Authors:** Ren Li, Yasser Rezaeiyan, Tim Böhnert, Alejandro Schulman, Ricardo Ferreira, Hooman Farkhani, Farshad Moradi

**Affiliations:** 1 Department of Electrical and Computer Engineering, Aarhus University, 8200 Aarhus, Denmark; 2 INL—International Iberian Nanotechnology Laboratory, 4715-330 Braga, Portugal

**Keywords:** Electrical and electronic engineering, Magnetic devices, Electronic devices

## Abstract

In this work, we present fabricated magnetic tunnel junctions (MTJs) that can serve as magnetic memories (MMs) or vortex spin-torque nano-oscillators (STNOs) depending on the device geometry. We explore the heating effect on the devices to study how the performance of a neuromorphic computing system (NCS) consisting of MMs and STNOs can be enhanced by temperature. We further applied a neural network for waveform classification applications. The resistance of MMs represents the synaptic weights of the NCS, while temperature acts as an extra degree of freedom in changing the weights and TMR, as their anti-parallel resistance is temperature sensitive, and parallel resistance is temperature independent. Given the advantage of using heat for such a network, we envision using a vertical-cavity surface-emitting laser (VCSEL) to selectively heat MMs and/or STNO when needed. We found that when heating MMs only, STNO only, or both MMs and STNO, from 25 to 75 °C, the output power of the STNO increases by 24.7%, 72%, and 92.3%, respectively. Our study shows that temperature can be used to improve the output power of neural networks, and we intend to pave the way for future implementation of a low-area and high-speed VCSEL-assisted spintronic NCS.

## Introduction

Conventional computers have been suffering from faster CPU and slower memory, known as the memory wall problem, and constant data fetching between memory and CPU further exacerbated the von Neumann bottleneck with large energy consumption^1^. With complementary metal–oxide–semiconductor (CMOS) scaling facing fundamental physics and economic roadblocks, von Neumann computers are also coming to an inevitable end in the near future^2^. In recent years, brain-inspired neuromorphic computing systems (NCS) have attracted research interest due to their massive parallel data processing capability and area- and power-efficient characteristics^3,4^. Spin-based electronics, or spintronics, are a promising technology to implement NCS by utilizing the extra degree of freedom of the intrinsic spin of electrons and their associated magnetic moment, in addition to their charge^5^. Spintronic devices exploit the coactions between the magnetic and electrical properties of materials to realize the characteristics needed for neuromorphic computing including nonvolatility and nonlinearity^6^. Magnetic tunnel junctions (MTJs) as the pivotal component in spintronics, have widely attracted research interests in using them as synapses and neurons in neuromorphic computing^7–10^. In particular, the temperature dependence of the resistance of MTJs has been reported for different material stacks^11–13^, indicating potential gain from the temperature effect. In this work, we investigate the benefit of temperature effect by using VCSEL on a neural network made of MTJs. We use two types of spintronic devices made by MTJs to mimic synapses and neurons. The spintronic synapse is implemented using nonvolatile magnetic memory (MM), where the synaptic weight is represented by its resistance value, and the neuron is represented by a nonlinear vortex spin-torque nano-oscillator (STNO).

## Results and discussion

### Auto-oscillation of vortex STNO

**Figure 1:**
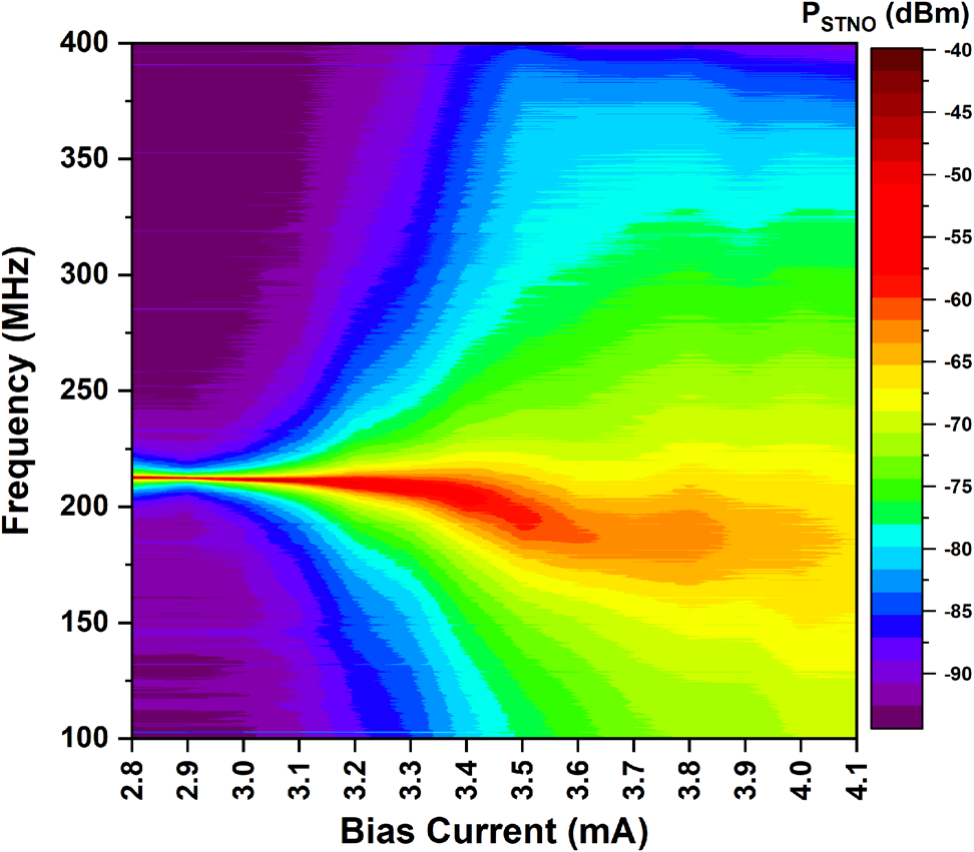
Measured vortex STNO output spectrum for different bias currents at room temperature; the in-plane field is − 54 Oe.

Figure 1 shows the measured oscillation spectrum of the 300 nm vortex STNO output at different bias currents at room temperature with an in-plane magnetic field of − 54 Oe. Note that the power shown here is the directly measured result from the spectrum analyzer; therefore, it is in dBm. From the plot, one can find some interesting observations. The peak oscillation frequency starts from 212.6 MHz and gradually decreases with increased bias current, this is expected as a larger bias current tends to excite a vortex oscillation with a larger orbit, which as a result reduces its oscillation frequency and increases its 3 dB linewidth. The peak oscillation power is − 49.9 dBm at 2.8 mA bias and gradually decreases with increased bias, one of the reasons behind it could be the device being closer to breakdown due to increased Joule-Heating at higher biasing current, therefore, the quality of the oscillator is worsened, and it has lower spin polarization and becomes noisier. It is noteworthy to point out that a more commonly used merit of STNO output power in circuit applications is the integrated power over the 3 dB linewidth range (also known as the full width at half maximum, FWHM), which is different from the peak oscillation power in Fig. 1, and it will be introduced in the next paragraphs together with the temperature effect tests.

### Temperature effect on STNOs and MMs

Thermally-induced or assisted neuromorphic systems have attracted research focus on various devices due to their beneficial effect^14–18^. In this work, we study the temperature effect on a spintronic neural network implemented using STNOs as neurons and MM as synapses.

#### Temperature effect on STNOs

**Figure 2:**
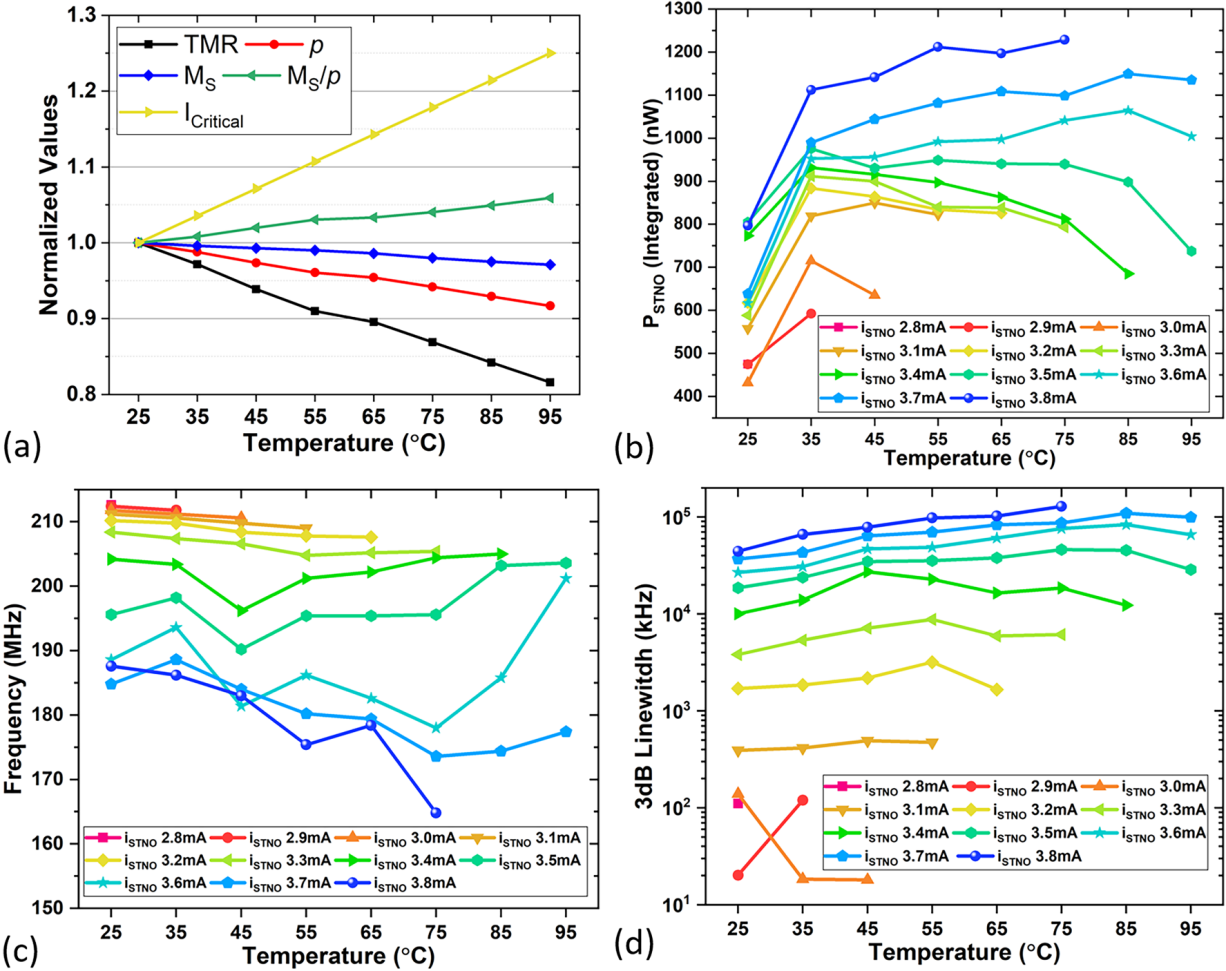
Measurement results for various aspects of the STNO versus temperature at different bias currents with an in-plane field of − 54 Oe: (**a**) temperature dependence of critical current, TMR, *p*, M_S_, and M_S_/*p* (**b**) output power, (**c**) peak oscillation frequency, and (**d**) the 3 dB linewidth.

We first studied the output of the vortex oscillator, where the applied in-plane magnetic field was − 54 Oe for all STNO measurements. We noticed that the critical current of the STNOs, namely, the minimum biasing current value for it to oscillate, increases linearly with temperature, as shown in Fig. 2a. The critical current at 25 **°**C is 2.8mA. This phenomenon can be understood by using the analytical expression of critical current from Dussaux et al*.*^19^, with Julliere’s model^20^ describing the spin polarization and the decrease in saturation magnetization with temperature^21^, we believe that the critical current should be approximately proportional to the behaviour of M_S_/*p* as a function of temperature. Based on the literature, here we show the temperature dependence of essential parameters in a normalized way in Fig. 2a including tunnel magnetoresistance TMR, spin polarization *p*, saturation magnetization M_S_, M_S_/*p*, and the measured critical current. Note that M_S_ is measured by vibrating-sample magnetometer (VSM) and we use measured values of TMR and M_S_ as a function of temperature in this estimation. The deviation between measured critical currents and theoretically predicted values might be an indication that it is related to a second mechanism that prevents the vortex from oscillation such as local pinning sites^22^. We then studied the effect on the oscillator output power. Figure 2b shows the STNO power (P_STNO_) versus temperature for different biasing conditions. P_STNO_ is the integrated value from the Lorentz fit of the obtained spectrum, as shown in Fig. 1, on a linear scale over its own FWHM range. The resolution bandwidth (RBW) used throughout the measurement is 50 kHz.

At each temperature, the output power almost always increases with higher biasing currents due to the resistance oscillation induced by vortex oscillation, which results in higher output power. Looking across different temperatures, from 25 to 35 **°**C, the oscillation power increases for all biasing conditions. From 35 **°**C and above, the oscillation power starts to decrease with temperature for i_STNO_ from 2.8 up to 3.4 mA, which could be because of the saturation of the oscillation trajectory. Starting from 3.5 mA and higher biasing currents, the decrease in output power comes at higher temperatures; for example, a significant decrease in power starts at 85 **°**C for the 3.5 mA biasing condition, 95 **°**C for 3.6 mA, and above 100 **°**C for 3.7 mA i_STNO_. A further increase in i_STNO_ leads to the trajectory of the vortex core being too large as it has exceeded the physical boundary of the 300nm diameter device and it was not able to be detected by the spectrum analyzer; therefore, we stopped the i_STNO_ increase at 3.8 mA. The same reason applies to no oscillation found with a 3.8 mA bias at higher temperatures above 75 **°**C. Overall, with certain biases in a certain range of temperature, for example, for i_STNO_ of 3.6 to 3.7 mA and the temperature range of 25 to 85 **°**C, the oscillation power increases for higher temperatures, which could be because of a transition between two grains that is favorable for the power, and this can be quite useful regarding a neural network implementation which will be discussed later. We summarize the relationship between the STNO output power, temperature, and biasing current in Table 1.

**Table 1:** Summary of the temperature effect on the STNO output power with respect to the biasing current.

	25–35 °C	35–85 °C	85–95 °C
2.8–3.4 mA	Increase	Decrease	N/A
3.5 mA	Increase	Constant	Decrease
3.6–3.8 mA	Increase	Increase	Decrease

In Fig. 2c and d, the peak oscillation frequency and the 3 dB linewidth of the oscillation signal are plotted against temperature for different biasing conditions. At higher biases, the STNOs have a wider trajectory, this larger orbit leads to a lower peak oscillation frequency and this negative slope in the frequency response is also confirmed by a prior work^19^ and we also observed a larger 3 dB linewidth. At higher temperatures, the oscillation frequency tends to decrease while its 3 dB linewidth increases.

#### Temperature effect on MMs

**Figure 3:**
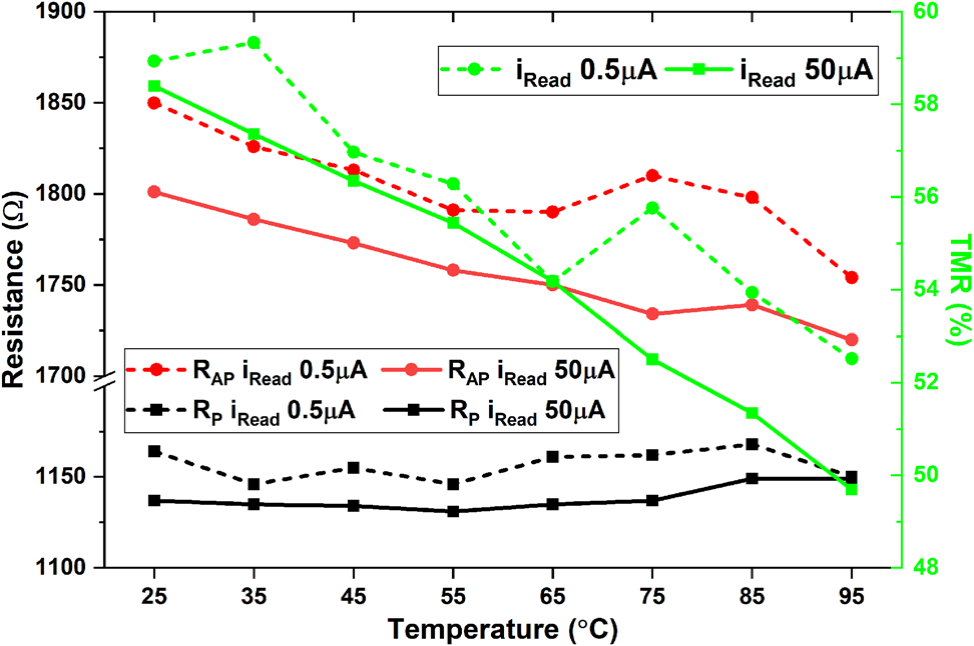
Measurement results of MM parallel and anti-parallel resistance and TMR when using a magnetic field to switch versus temperature for two read currents.

We then studied the temperature effect on magnetic memory, as shown in Fig. 3. The parallel and anti-parallel states are represented by R_P_ and R_AP_ with their values obtained by performing a sweep on the in-plane magnetic field. The tunnel magnetoresistance (TMR) is denoted in green, which corresponds to the axis on the right. TMR decreases at higher temperatures, making the device less useful for storing information; however, the change in R_AP_ makes it more attractive for neuromorphic computing. We notice that the parallel resistance (R_P_) changes slightly with temperature, while the anti-parallel resistance (R_AP_) decreases with increased temperature, this could be a result of the competition between ohmic and defect-driven semiconductor-alike behavior^12^. And this behavior could be particularly useful in neural networks as R_P_ is constant and R_AP_ can be used for implementing synaptic weights. Here, the weight is represented by its conductance value (G_AP_), and it increases with temperature; namely, we could use heat as an extra degree of freedom to adjust the weight. To verify that this change in resistance is not purely caused by Joule heating from the read current, we also performed the measurement with only 1% of the normal read current, 0.5 µA, and the observations remained the same as shown in Fig. 3. When building a neural network with MMs as synapses, increasing the temperature will lead to more current flowing into the connected neurons; this temporary enhancement of the postsynaptic response is known as short-term facilitation (STF)^23^ due to the short-term plasticity (STP) of the neural network^24^, and potential benefits will be analyzed in the next paragraphs.

### Neural network implementation and analysis

**Figure 4:**
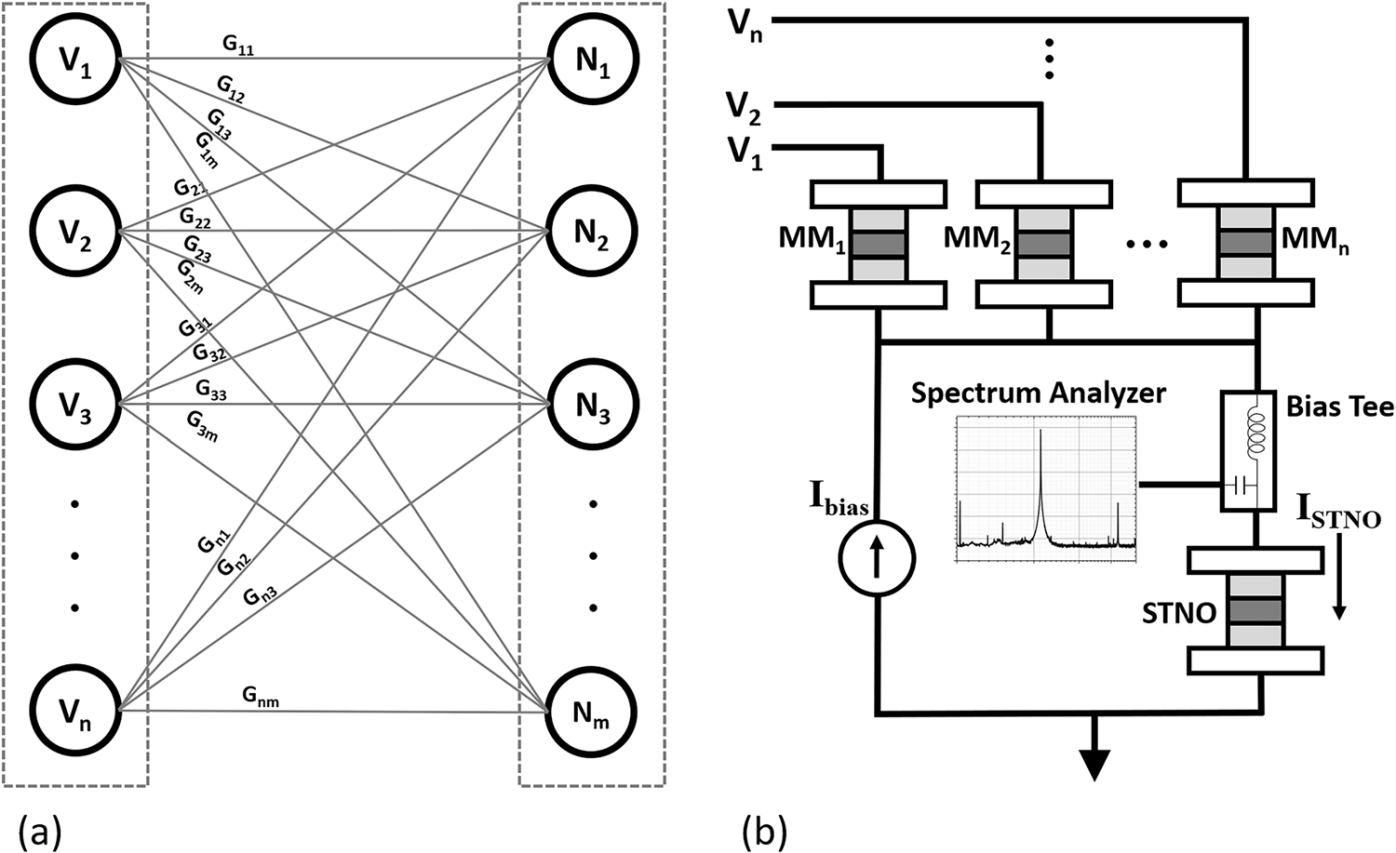
(**a**) Schematic of a neural network with two neural layers being connected by synapses with different weights represented by P or AP states of MMs (**b**) Implementation of a neural network consisting of multiple MMs and one STNO for analysis.

We envision a neural network consisting of MMs and STNOs as drawn in Fig. 4a, with STNOs as neurons in different layers and MMs as the synapses connecting pre- and postsynaptic neurons in the two layers. The synaptic weights are represented by the P or AP states of the MM, and G_nm_ indicates the electrical denotation of the synaptic weight in conductance. According to the total summation of the weights, a neuron in the next layer might fire or not, depending on whether its critical current level has been reached or not. In the following analysis, we use one STNO and fifty MMs as an example to investigate the temperature effect on a neural network, as shown in Fig. 4b. During the measurement, we used Peltier as a hot plate to heat the sample; in the future, we intend to use a vertical-cavity surface-emitting laser (VCSEL) array for heating purposes due to a higher level of integration, potential for low power consumption, and capability for easier and faster training. For example, we could design a VCSEL array with individual access to each laser beam to selectively heat a certain part of the chip. According to the COMSOL simulation presented in a prior work^16^ on VCSEL heating for a 350 nm diameter MTJ, by calculating the average laser power based on the device size and the targeted temperature, we estimate the worst-case scenario laser power consumption for the analysis in the rest of this section. Here we summarize the findings of the gain in output power when heating different parts of the sample in Table 2 for three different scenarios:

**Table 2:** Summary of the power analysis for heating various parts of the chip from 25 to 75 **°**C.

	Scenario I: heat MMs Only	Scenario II: heat STNO Only	Scenario III: heat MMs and STNO
ΔP_STNO_ (nW)	158	460	590
ΔP_STNO_ (%)	24.7	72	92.3

#### Scenario I

Assuming that all fifty MMs are identical and set to anti-parallel states, when we selectively heat the MMs from 25 to 75 **°**C while keeping the STNO at 25 **°**C, according to Fig. 3, the resistances of MMs would change from 1801 to 1734 Ω. With the same supply voltage on the MMs, for instance, a 0.1 V voltage difference between the two terminals of each MM; by increasing the temperature to 75 **°**C, one can expect an extra 0.1 mA current to flow into the STNO. When we choose a constant external bias current source I_bias_ of 0.92 mA, the total current flowing into the STNO changes from 3.7 to 3.8 mA, which leads to an extra 24.7% (158 nW) oscillation power at the STNO output. Based on the COMSOL simulation from a previous work^16^, we estimate that it would cost 879 µW (COMSOL Simulation) of laser power to heat fifty MMs to 75 **°**C.

#### Scenario II

When we keep the MMs at 25 **°**C and only heat the STNO, for example, from 25 to 75 **°**C, with the total current flowing into the STNO, I_STNO_, being 3.7 mA, according to Fig. 2b, one can expect an extra 72 % (460 nW) output power, compared to the case at 25 **°**C. It is estimated that heating the 300 nm STNO to 75 **°**C would consume 23.5 µW (COMSOL Simulation) of laser power.

#### Scenario III

If we heat both MMs and STNO from 25 to 75 **°**C, the result is effectively a combination of heating STNO to 75 **°**C and increasing its total current I_STNO_ from 3.7 mA to 3.8 mA, according to Fig. 2b, it will give us 92.3 % (590 nW) extra output power compared to the case of 3.7 mA at 25 **°**C, and it would consume 902 µW (COMSOL Simulation) for heating. An increase in the output power of the oscillators will also ease the design requirement on the following CMOS readout circuit, as there is a linear relationship between the power consumption of a receiver and its sensitivity^25^.

The increased output power of STNO leads to an increase in the SNR of the signal, as higher SNR needs lower sensitivity, one can say when the output power of the STNO increases 1.92 times, the power consumption of the CMOS part would decrease to 0.52 times, leading to a 3.54mW power saving for the whole system. Thus, an increase in STNO output power also helps to reduce the total power consumption of the system.

For all three scenarios, the gain at the output is less than the estimated power consumed by the laser at the input. This is due to the fact that the energy conversion efficiency of the system is still low; however, the ratio of increment is promising, as heating MMs and the STNO can increase the output power up to 92%. We expect VCSEL to consume less power while delivering the same amount of heat in future development especially if a lens is added on top of the spintronic devices to lower the light loss. With the improved MTJ composition and fabrication, we also expect the STNO to have higher energy conversion efficiency and larger output power. Moreover, we plan to fabricate MMs with a lower resistance so that each MM can deliver more current, and a smaller number of MMs are needed for the network; thus, less heating is needed for temporarily changing the weights, and we could also eliminate the need for an external biasing current source for the network. Overall, we have seen through measurement that increasing the temperature can help the neural network gain more output power. We anticipate that by collaboratively improving material development, device composition and fabrication process optimization, and advancing optical components, we could have a neuromorphic computing system with improved energy conversion efficiency.

### Application in waveform classification

To demonstrate the feasibility of using such a neuromorphic system, we simulated a neuromorphic network consisting of 3 STNO neurons with 100 MM weights for waveform classification applications in Cadence Virtuoso. We digitize sinusoidal, triangular, pulse, and their inverted waves to 10 × 10 pixels pictures and included the temperature effect into our custom STNO model. We observed correct functionality of the system, more details on the proposed waveform classification application along with simulation results are described as follows.

**Figure 5:**
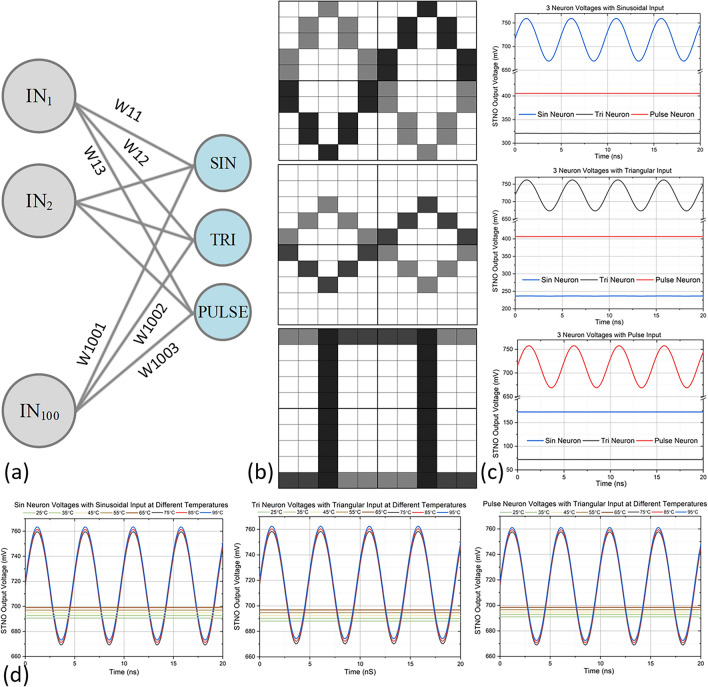
(**a**) The schematic of the proposed waveform classification neural network, the biasing current for each neuron is different from each other. (**b**) The pixelized representation of the sinusoidal, triangular, pulse and their inverted waveforms. (**c**) The STNO output voltage of all three neurons at 75 °C with different input patterns, indicates that the dedicated neuron only fires when the corresponding waveform is the input. (**d**) The SNTO output voltage of each neuron from 25 to 95 °C, showing that the neuron fires starting from 75 °C and the output increases with temperature.

The proposed weighted neural network consisting of 100 MMs and 3 STNOs is shown in Fig. 5a for waveform classification applications. Each of the 3 STNOs represents the neuron that classifies sinusoidal, triangular, and pulse waves, later referred to as sin, tri, and pulse neurons. We train our neural network to classify the most frequently used sinusoidal, triangular, pulse waves and their inverted shapes. For the classification task, we digitize the input waveforms into 10 × 10 pixels pictures using a 1-bit digital-to-analog converter (DAC). The converted outputs are high (digital “1”) or low (digital “0”) voltage signals feeding directly to the neural network, along with their inverted waveforms, they are denoted by black or gray respectively as digital “1” and white as digital “0” in a pixel, as shown in Fig. 5b. These voltage signals are then translated to current signals that bias the STNOs based on the weights of the network, where we use the temperature-sensitive anti-parallel resistance of MM as the strong (digital “1”) connection and open circuit to represent the weak (digital “0”) connection.

According to the circuit equivalent of the proposed neural network in Fig. 4b, it consists of voltage sources and current source, therefore, according to Thévenin's theorem, one can derive to the below relationship:

1 $${I}_{STNO}=\left(\sum {V}_{i}{G}_{i}+{I}_{Bias}\right)\times \frac{{G}_{STNO}}{\sum {G}_{i}+{G}_{STNO}}.$$

With I_STNO_ being the total excitation current for STNO, V_i_ and G_i_ being the input voltage and conductance of each MM, I_Bias_ being the extra current source providing biasing current.

The MMs weight and accumulate the inputs, which are then nonlinearly converted into the output power P_STNO_ governed by the following equation^9^:

2 $$y=f\left(\sum {V}_{i}{G}_{i}+{I}_{Bias}\right).$$

With f being the non-linear transfer function between the input current and output power of STNO.

Each STNO corresponds to a waveform and based on the weights that we assigned to the network according to the above equations; we guarantee that only one of the STNOs oscillates for each input waveform, the correct functionality of the proposed neural network is shown in Fig. 5c. We also added the temperature effect from measurement data to our custom STNO and MM model and simulated the STNO output voltages for each input pattern using Cadence Virtuoso Spectre^®^, verifying the temperature effect of the proposed neural network, as shown in Fig. 5d, the neuron fires starting from 75 °C and the output increases with temperature, same as what was observed in the measurement.

## Conclusions

In this work, the temperature effect of MTJ-based MMs and STNOs has been experimentally demonstrated and analyzed for neuromorphic computing applications. We found that the performance of an NCS, namely, the output power of the STNO, can be enhanced by up to 92.3% due to the heating effect. We implemented a neural network for waveform classification applications where we can classify sinusoidal, triangular, and pulse waves. In the future, we plan to use VCSEL for heating purposes, as it gives an extra degree of freedom in changing the resistance value and TMR of MMs; thus, the weights of an NCS, and VCSEL can also be used for easier and faster training purposes. With the improvement in device fabrication to boost the STNO energy conversion efficiency and advancement in photonics to reduce VCSEL power consumption, we envision a future implementation of low-area and high-speed VCSEL-assisted spintronic neuromorphic computing systems.

## Methods

### MTJ-based magnetic memory and spin-torque nano-oscillator

**Figure 6:**
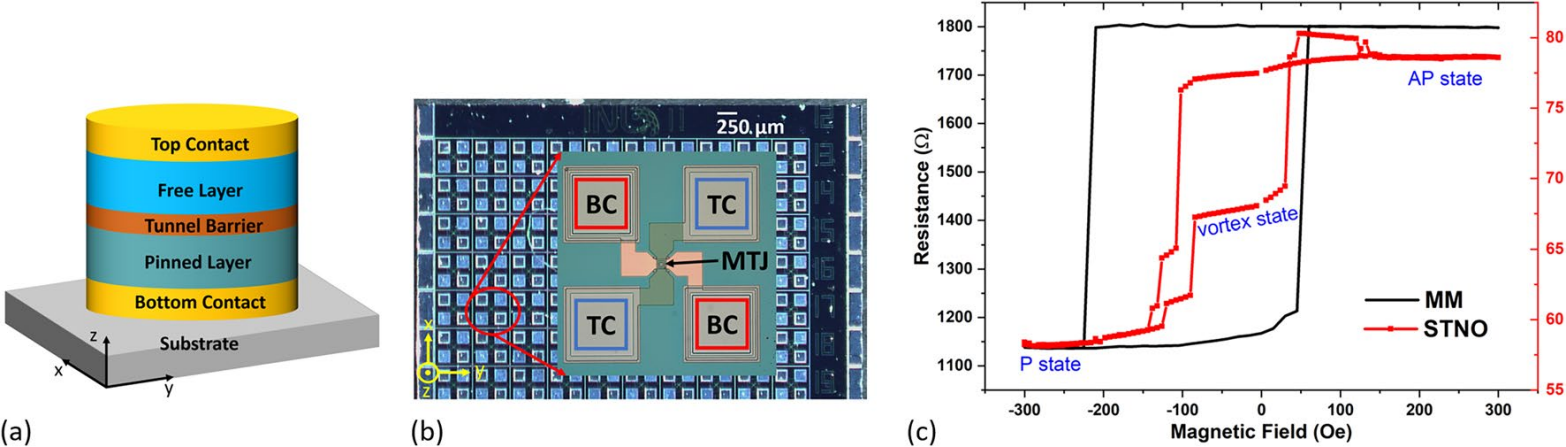
(**a**) Schematic of spintronic devices made by MTJ (**b**) Optical microscopy image of fabricated die. The insert shows an enlarged structure of one MTJ with top contacts (TC) and bottom contacts (BC). (**c**) Transfer curve of resistance versus magnetic field of MM in black and STNO in red. The reading current used is 50 µA.

The schematic structure of the spintronic devices used in this work is shown in Fig. 6a, which contains the bottom contact, the MTJ stack consisting of a pinned layer, a tunnel barrier, and a free layer, and the capping structure as the top contact. The deposition of material is achieved by magnetron sputtering, followed by annealing, and through patterning using e-beam lithography and ion-beam milling, a nanopillar is formed^26^. The bottom contact is formed by 5 Ta/50 CuN/5 Ta/50 CuN/5 Ta/5 Ru, the top contact is made of 10 Ta/7 Ru, and the MTJ stack contains 6.0 Ir_0.2_Mn_0.8_/2.0 Co_0.7_Fe_0.3_/0.7 Ru/2.6 Co_0.4_Fe_0.4_B_0.2_/0.8 MgO/2.0 Co_0.4_Fe_0.4_B_0.2_/0.2 Ta/7.0 NiFe (thickness in nanometers). The MgO layer serves as the tunneling barrier and defines the resistance-area (R × A) product of the MTJ. The 6.0 Ir_0.2_Mn_0.8_/2.0 Co_0.7_Fe_0.3_/0.7 Ru/2.6 Co_0.4_Fe_0.4_B_0.2_, known as synthetic antiferromagnetic, which acts as the pinned layer, and the 2.0 Co_0.4_Fe_0.4_B_0.2_/0.2 Ta/7.0 NiFe layer is the free layer. After annealing, the free layer will have an easy axis along the orthogonal in-plane direction of the sample, namely, the y-axis in Fig. 6a. An optical photo of the fabricated die is shown in Fig. 6b with top and bottom contacts and MTJ annotated. Note that the MM and STNO used in this work are fabricated separately from different batches with the same MTJ stack composition. The diameter of the nanopillar determines the characteristics of the device. An elliptical-shaped device with small diameters tends to have more stable parallel and antiparallel states that are suitable to be used as MM, and a circular-shaped device with a certain diameter provides vortex states that can then be used as STNO^9^ The MMs used in this work are elliptical nanopillars with a diameter of 75 nm on the x-axis and 225 nm on the y-axis, and the STNOs are circular nanopillars with 300 nm in diameter. Their characteristics are shown by the resistance versus external in-plane magnetic field plot in Fig. 6c, respectively, with MM showing two distinct states indicating the parallel (P) and antiparallel (AP) states, and STNO showing intermediate states in between the P and AP states as vortex states.

### Measurement process

**Figure 7:**
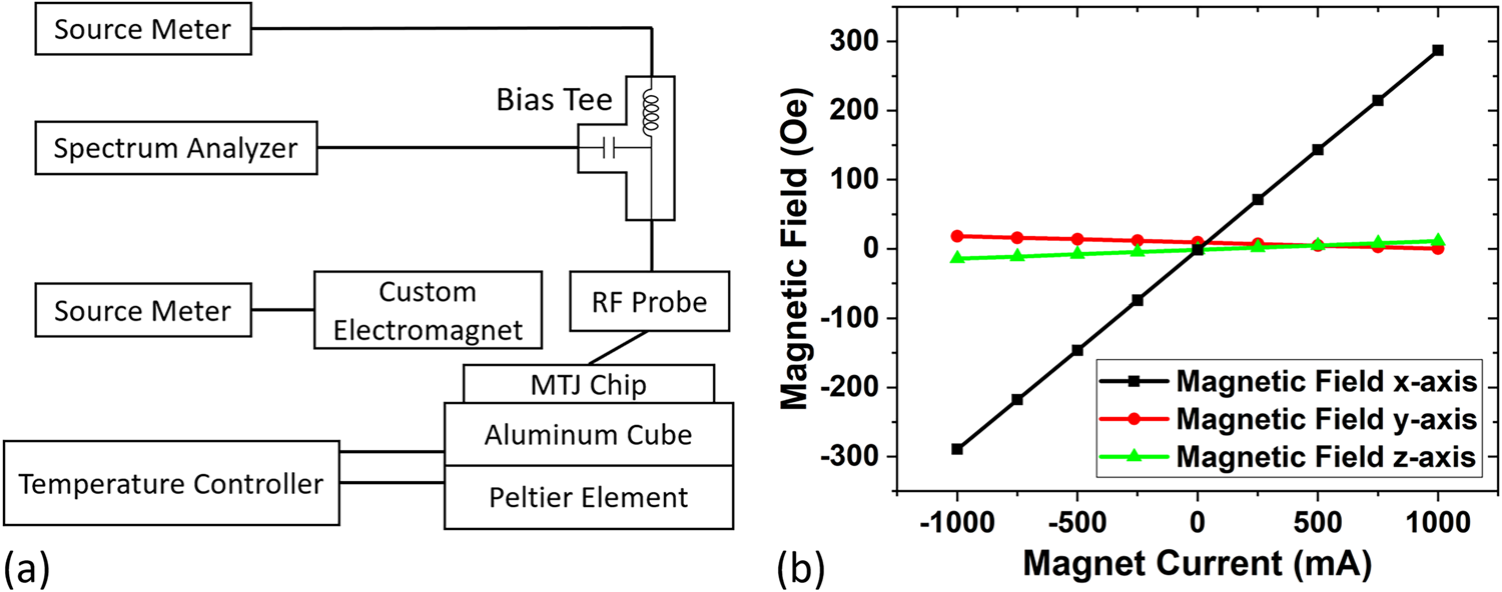
(**a**) Schematic of the measurement setup (**b**) Magnetic field versus applied current of the custom electromagnet, measured by a GMW 3MTS magnetic sensor placed at the center of the opening.

A schematic of the measurement setup used in this work is shown in Fig. 7a; the actual setup with an illustration can be found in Supplement Video S1. We use two Keithley source meters to supply biasing current to the device and control the custom-built electromagnet, respectively. We use the Thorlabs temperature controller to study the temperature effect on devices, and a Rohde & Schwarz spectrum analyzer to acquire the RF signal generated by the STNO output. The sample is mounted on top of a custom aluminum (Al) cube and a Peltier element. The Peltier is used for heating the device, and the thermistor positioned at the inner middle of the Al cube is used for sensing the temperature. The communication between Peltier, the thermistor, and the temperature controller is realized through a custom circuit board. A high-performance RF probe is used for probing the device, together with a Bias Tee, connecting the device under test, a source meter, and the spectrum analyzer together while isolating the DC and RF components. A custom-built electromagnet with a 1 cm opening is used for generating the in-plane magnetic field, and Fig. 7b shows its magnetic field versus the applied current of the custom electromagnet. To find the oscillation of the STNO, we first obtain a transfer curve by sweeping the magnetic field and measuring the device resistance by applying a small reading current, as shown in Fig. 6c, to verify the validity of the device and determine the vortex states. Then, a biasing current equal to or above the critical current is applied to nucleate the vortex. This is achieved by saturating the device in either the P or AP direction and slowly reducing the magnetic field to facilitate vortex renucleation. Subsequently, the magnetic field is swept in smaller steps within the vortex range, while monitoring the spectrum analyzer to obtain auto-oscillation^27^.

### Supplementary Information

Supplementary Video S1.

## Data Availability

The data that support the findings of this study are available within the article, and they are from the corresponding author upon reasonable request.
